# Risk factors for diabetic kidney disease in type 2 diabetes mellitus in Asia: a meta-analysis

**DOI:** 10.3389/fendo.2026.1703533

**Published:** 2026-04-14

**Authors:** Yaning Zheng, Sheng Ma, Yong Chen, Cairong Li

**Affiliations:** 1 Department of Nephrology, Xianning Central Hospital, First Affiliated Hospital of Hubei University of Science and Technology, Xianning, China; 2 Department of Anorectal, Xianning Central Hospital, First Affiliated Hospital of Hubei University of Science and Technology, Xianning, China; 3 Medical College, Xianning Medical College, Hubei University of Science and Technology, Xianning, China

**Keywords:** complications, DKD, meta- analysis, risk factors, T2DM

## Abstract

**Background:**

Diabetic kidney disease (DKD) is a highly significant microvascular complication that arises from diabetes. Therefore, this study aimed to ascertain the traditional risk factors for DKD in type 2 diabetes mellitus (T2DM) in Asia, raising awareness of these risk factors among patients with T2DM.

**Methods:**

PubMed, Embase, Web of Science, and Cochrane Library were systematically searched until 13 Mar 2026. Case–control or cohort studies in Asia on the risk factors for DKD were included. Egger’s test and funnel plots were used to assess publication bias. Stata 15 was used for statistical analysis.

**Results:**

7 case-control studies (including 3,312 participants) and 17 cohort studies (including 8,735 participants) were included. All the included studies were of high quality according to the Newcastle-Ottawa Scale (NOS). Systolic blood pressure (SBP), hypertension, glycosylated hemoglobin (HbA1c), waist-to-hip ratio (WHR), fasting blood glucose (FBG), uric acid (UA), creatinine (Cr), age and diabetes duration were risk factors for DKD in T2DM. Diabetic retinopathy (DR) was closely associated with DKD, and this association was also evident in subgroups defined by pathological diagnosis. SBP was a risk factor in both the clinical diagnosis group and the pathological diagnosis group.

**Conclusions:**

This meta-analysis preliminarily demonstrates that SBP, hypertension, HbA1c, WHR, FBG, UA, Cr, age, diabetes duration and DR are associated with DKD in Asia. SBP and DR are associated with renal biopsy-confirmed DKD.

**Systematic review registration:**

https://www.crd.york.ac.uk/PROSPERO/recorddashboard, identifier CRD42024529789.

## 1 Introduction

T2DM, caused by the prolonged interplay of genetic and environmental factors, is characterized by insufficient insulin secretion and insulin resistance, accounting for more than 90% of diabetes cases (1, 2). It is typically manifested as polydipsia, polyphagia, polyuria, weight loss, and fatigue (2). In 2021, about 529 million people were afflicted by diabetes globally, and this figure may rise to more than 1.31 billion by 2050 (3). About 20%-40% of diabetes cases may progress into DKD (4). DKD, as a microvascular complication of diabetes, can cause end-stage renal disease (ESRD) (4, 5). The incidence of ESRD events caused by diabetes has increased from 22.1% to 31.3% worldwide (6). Based on the GBD data from 1990 to 2023, there were approximately 4.59 million cases of kidney failure with replacement therapy (KFRT) worldwide in 2023, with the primary causes including type 2 diabetes and hypertension (7). Therefore, it is crucial to explore the risk factors for DKD.

DKD is attributable to chronic hyperglycemia and affects the entire kidney, including glomeruli, renal tubules, tubulointerstitium, and renal blood vessels. The primary pathological features of DKD are diffuse thickening of the glomerular basement membrane, expanded glomerular mesangium, Kimmelstiel-Wilson (K-W) nodules, fusion of podocyte foot processes and glomerular sclerosis, exudative lesions (such as renal capsule drops and cellulose caps), tubular atrophy and interstitial fibrosis, inflammatory cell infiltration, and hyalinization of renal arterioles. It is clinically manifested as persistent albuminuria and/or a progressive decline in estimated glomerular filtration rate (eGFR), such as urinary albumin-to-creatinine (UACR) ≥ 30 mg/g and/or estimated glomerular filtration rate (eGFR) < 60 mL·min^-1^·(1.73 m²)^-1^, persistent for at least 3 months, with renal diseases of other causes excluded in KDIGO (8, 9). However, non-diabetic kidney disease (NDKD) occurs in patients with clinically diagnosed DKD. Pathological diagnosis is the gold standard for diagnosing DKD, which is confirmed by renal biopsy. Currently, the risk factors for DKD can be categorized into susceptibility factors (such as age, gender, race, and obesity), initiation factors (such as hyperglycemia and genetic triggers), and progression factors (such as hypertension, smoking, and renal toxicity) (10). The most prominent accepted risk factors for DKD encompass hypertension, hyperglycemia, and obesity. Many studies have explored risk factor-based models for predicting DKD in T2DM patients in clinical settings (11–13). Although some reviews have reported risk factors (such as age, obesity, a history of T2DM, and hypertension) for DKD, there is still a lack of quantitative analysis from different data sources for risk factors in different diagnostic patterns (14, 15). Therefore, to ensure the reliability of the results, we clearly defined DKD and conducted a meta-analysis of risk factors after classifying populations based on their diagnostic patterns.

This meta-analysis aims to unveil traditional risk factors for DKD in Asia and hopes to improve the prognosis and quality of life of affected individuals.

## 2 Materials and methods

This meta-analysis was reported in accordance with the PRISMA guidelines and was registered on PROSPERO (CRD42024529789).

### 2.1 Literature search

Relevant studies published in English were searched from Cochrane Library, PubMed, Embase, and Web of Science until 13 Mar 2026. The search terms were designed by combining subject terms and free words. The keywords involved diabetes mellitus, type 2, diabetic nephropathies, and risk factors. The specific search strategy for PubMed is available in **Table 1**.

**Table 1:** Search strategy of Pubmed.

Search query	
#1	(Diabetes Mellitus, Type 2)[MeSH Terms]
#2	(Diabetes Mellitus, Type 2[Title/Abstract]) OR (Diabetes Mellitus, Noninsulin-Dependent[Title/Abstract])) OR (Diabetes Mellitus, Ketosis-Resistant[Title/Abstract])) OR (Diabetes Mellitus, Ketosis Resistant[Title/Abstract])) OR (Ketosis-Resistant Diabetes Mellitus[Title/Abstract])) OR (Diabetes Mellitus, Non Insulin Dependent[Title/Abstract])) OR (Diabetes Mellitus, Non-Insulin-Dependent[Title/Abstract])) OR (Non-Insulin-Dependent Diabetes Mellitus[Title/Abstract])) OR (Diabetes Mellitus, Stable[Title/Abstract])) OR (Stable Diabetes Mellitus[Title/Abstract])) OR (Diabetes Mellitus, Type II[Title/Abstract])) OR (NIDDM[Title/Abstract])) OR (Diabetes Mellitus, Noninsulin Dependent[Title/Abstract])) OR (Diabetes Mellitus, Maturity-Onset[Title/Abstract])) OR (Diabetes Mellitus, Maturity Onset[Title/Abstract])) OR (Maturity-Onset Diabetes Mellitus[Title/Abstract])) OR (Maturity Onset Diabetes Mellitus[Title/Abstract])) OR (MODY[Title/Abstract])) OR (Diabetes Mellitus, Slow-Onset[Title/Abstract])) OR (Diabetes Mellitus, Slow Onset[Title/Abstract])) OR (Slow-Onset Diabetes Mellitus[Title/Abstract])) OR (Type 2 Diabetes Mellitus[Title/Abstract])) OR (Noninsulin-Dependent Diabetes Mellitus[Title/Abstract])) OR (Noninsulin Dependent Diabetes Mellitus[Title/Abstract])) OR (Maturity-Onset Diabetes[Title/Abstract])) OR (Diabetes, Maturity-Onset[Title/Abstract])) OR (Maturity Onset Diabetes[Title/Abstract])) OR (Type 2 Diabetes[Title/Abstract])) OR (Diabetes, Type 2[Title/Abstract])) OR (Diabetes Mellitus, Adult-Onset[Title/Abstract])) OR (Adult-Onset Diabetes Mellitus[Title/Abstract])) OR (Diabetes Mellitus, Adult Onset[Title/Abstract])
#3	(Diabetic Nephropathies)[MeSH Terms]
#4	(Diabetic Nephropathies[Title/Abstract]) OR (Nephropathies, Diabetic[Title/Abstract])) OR (Nephropathy, Diabetic[Title/Abstract])) OR (Diabetic Nephropathy[Title/Abstract])) OR (Diabetic Kidney Disease[Title/Abstract])) OR (Diabetic Kidney Diseases[Title/Abstract])) OR (Kidney Disease, Diabetic[Title/Abstract])) OR (Kidney Diseases, Diabetic[Title/Abstract])) OR (Diabetic Glomerulosclerosis[Title/Abstract])) OR (Glomerulosclerosis, Diabetic[Title/Abstract])) OR (Intracapillary Glomerulosclerosis[Title/Abstract])) OR (Nodular Glomerulosclerosis[Title/Abstract])) OR (Glomerulosclerosis, Nodular[Title/Abstract])) OR (Kimmelstiel-Wilson Syndrome[Title/Abstract])) OR (Kimmelstiel Wilson Syndrome[Title/Abstract])) OR (Syndrome, Kimmelstiel-Wilson[Title/Abstract])) OR (Kimmelstiel-Wilson Disease[Title/Abstract])) OR (Kimmelstiel Wilson Disease[Title/Abstract])
#5	(Risk Factors) [MeSH Terms]
#6	(Risk Factors[Title/Abstract]) OR (Factor, Risk[Title/Abstract])) OR (Risk Factor[Title/Abstract])) OR (Social Risk Factors[Title/Abstract])) OR (Factor, Social Risk[Title/Abstract])) OR (Factors, Social Risk[Title/Abstract])) OR (Risk Factor, Social[Title/Abstract])) OR (Risk Factors, Social[Title/Abstract])) OR (Social Risk Factor[Title/Abstract])) OR (Health Correlates[Title/Abstract])) OR (Correlates, Health[Title/Abstract])) OR (Population at Risk[Title/Abstract])) OR (Populations at Risk[Title/Abstract])) OR (Risk Scores[Title/Abstract])) OR (Risk Score[Title/Abstract])) OR (Score, Risk[Title/Abstract])) OR (Risk Factor Scores[Title/Abstract])) OR (Risk Factor Score[Title/Abstract])) OR (Score, Risk Factor[Title/Abstract])
#7	#1 OR #2
#8	#3 OR #4
#9	#5 OR #6
#10	#7 AND #8 AND#9

### 2.2 Screening criteria

Clinical diagnosis of DKD was defined as eGFR <60 mL/min/1.73 m^2^ or/and urinary albumin-to-creatinine (UACR) ≥30 mg/g (or albumin excretion rate [AER] ≥30 mg) for more than 3 months.

Pathological diagnosis of DKD was defined as DKD confirmed by renal biopsy.

Inclusion criteria were as follows: 1) populations: healthy individuals and T2DM patients (age ≥18 years) in Asia; 2) comparison/control: patients without DKD; 3) outcome: DKD (by clinical diagnosis or pathological diagnosis); 4) study type: case–control or cohort study (retrospective or prospective).

Exclusion criteria were as follows: 1) patients with T1DM; 2) patients with T2DM complicated by other renal diseases or acute complications; 3) animal experiments; 4) review, case report, meta-analysis, conference, and letter.

### 2.3 Data extraction

Two reviewers (Yaning Zheng and Sheng Ma) independently screened the searched studies. Inappropriate literature was eliminated by reading titles and abstracts, and the full texts were read to determine eligible studies. Any disputes were addressed through discussion with a third reviewer (Cairong Li). The following data were extracted: author, year, study design, country, sample size, number of DKD, mean age, relevant risk factors, regression model, and definition of DKD.

### 2.4 Quality assessment

The Newcastle-Ottawa Scale (NOS) was used to assess the quality of case–control studies and cohort studies (16–18) in the selection of study populations (4 points), comparability (2 points), and exposure factors or outcome measurements (3 points). The NOS score ranged from 0 to 9 stars. A NOS score of higher than 7 stars indicated high quality. Two researchers assessed the methodological quality independently. Disagreements were addressed through discussion with a third researcher.

### 2.5 Statistical analysis

Meta-analysis was conducted for risk factors reported in at least three studies. The included studies reported point estimates as odds ratios (ORs) with 95% confidence intervals (95% CIs). If no significant heterogeneity was observed (I^2^ < 50%, P ≥ 0.1), the fixed-effects model was utilized. Otherwise, the random-effects model was adopted (19). Sensitivity analysis was carried out by excluding each article. The publication bias was assessed using Egger’s test and funnel plots. Statistical analysis was performed using Stata 15, and P < 0.05 indicated statistical significance.

## 3 Results

### 3.1 Study screening and characteristics

**Figure 1:**
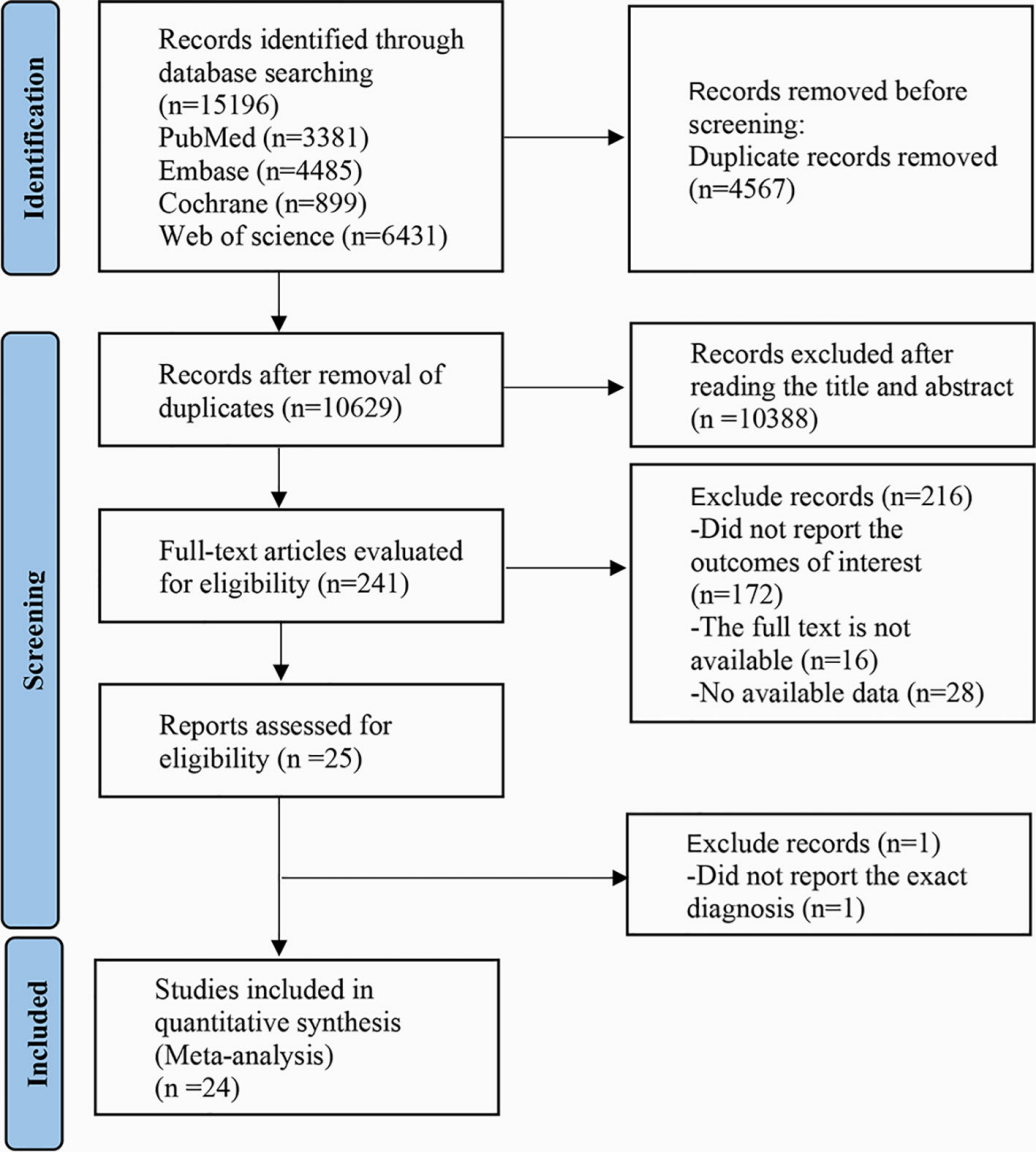
PRISMA flow diagram of the study process. PRISMA, preferred reporting items for systematic review and meta-analysis.

A total of 15,196 records were searched from databases. After removing duplicates, we screened the titles and abstracts of 10,388 publications. Then, we reviewed 241 full-text articles. Finally, 24 articles (20–43) with 12,056 participants were included. The detailed process of literature screening is displayed in **Figure 1**. The included articles were published between 2007 and 2025. The participants of the included studies were from Asia, including China, Japan, India, and Bangladesh. The basic characteristics are displayed in **Table 2**. There were 7 case-control studies and 17 cohort studies. The NOS scores of the included studies were all over 7, as shown in **Supplementary Table 1**.

**Table 2:** The basic characteristics of involved literature.

Study	Year	Study design	Country	Sample size	No. of DKD	Mean age (year)	Diagnose of DKD	Multiple regression model
T Li	2023	Case-control study	China	132	44	55.5-66.5	clinical diagnosis	logistic regression
XJ Ren	2023	Case-control study	China	243	116	57.78	clinical diagnosis	logistic regression
R Wang	2021	Case-control study	China	284	118	58.39	clinical diagnosis	logistic regression
QH Huang	2020	Case-control study	China	402	99	63	clinical diagnosis	logistic regression
XM Fei	2018	Case-control study	China	301	99	63	clinical diagnosis	logistic regression
YQ Huang	2024	Case-control study	China	141	35	55.86	clinical diagnosis	logistic regression
J Du	2025	Case-control study	China	1809	486	56.15	clinical diagnosis/pathological diagnosis	logistic regression
SY Duan	2020	Cohort study	China	100	79	52.48	pathological diagnosis	logistic regression
Furukawa	2014	Cohort study	Japan	513	29	61.1	clinical diagnosis	logistic regression
HY Huang	2019	Cohort study	China	115	48	60	clinical diagnosis	logistic regression
XY Wang	2019	Cohort study	China	220	126	54.7	pathological diagnosis	logistic regression
J Wei	2022	Cohort study	China	59	25	48.36	pathological diagnosis	logistic regression
DM Zhou	2022	Cohort study	China	102	52	51	pathological diagnosis	logistic regression
J Chen	2024	Cohort study	China	141	45	59.33	clinical diagnosis	logistic regression
Unnikrishnan	2007	Cohort study	Indian	1716	38	57	clinical diagnosis	logistic regression
Y Peng	2015	Cohort study	China	448	144	65	clinical diagnosis	logistic regression
J Xu	2021	Cohort study	China	402	206	59.19	clinical diagnosis	logistic regression
XJ Wang	2023	Cohort study	China	132	61	54.49	pathological diagnosis	LASSO regression
HF Li	2023	Cohort study	China	673	345	65	clinical diagnosis	logistic regression
AM Li	2019	Cohort study	China	209	44	66.4	clinical diagnosis	linear regression
SM Shi	2023	Cohort study	China	1142	368	58	clinical diagnosis	logistic regression
Afroz	2019	Cohort study	Bangladesh	1475	429	–	clinical diagnosis	logistic regression
Raman	2012	Cohort study	Indian	248	26	53.6	clinical diagnosis	logistic regression
ZL Deng	2025	Cohort study	China	1049	139	–	clinical diagnosis/pathological diagnosis	logistic regression

### 3.2 Meta-analysis of risk factors for DKD

A total of 15 related factors, including SBP, hypertension, FBG, HbA1c, UA, Cr, age, diabetes duration, DR, WHR, diastolic blood pressure (DBP), body mass index (BMI), blood urea nitrogen (BUN), male gender, and albumin (ALB) for DKD were analyzed (**Table 3**). Among them, 10 risk factors for DKD were identified.

**Table 3:** Summary of risk factors identified among included studies.

Risk factors	No. of study	heterogeneity		OR (95%CI)	P	Egger
		I^2^(%)	P			
SBP	11	45.6	0.049	1.02(1.02, 1.02)	0.000	0.051
Hypertension	5	56.4	0.057	2.58 (1.66, 4.02)	0.000	0.326
FBG	3	3.6	0.354	1.32 (1.14, 1.54)	0.000	0.378
HbA1c	5	93.8	0.000	1.22(1.04, 1.44)	0.015	0.047
UA	4	0.0	0.465	1.00 (1.00,1.01)	0.004	0.426
Cr	5	66.3	0.018	1.02 (1.00,1.04)	0.019	0.575
Age	7	2.0	0.410	1.02 (1.01, 1.03)	0.000	0.387
Diabetes duration	10	80.2	0.000	1.09 (1.04, 1.14)	0.000	0.105
DR	4	38.5	0.181	20.04(9.77,41.11)	0.000	0.434
WHR	3	0.0	0.390	3.53 (1.68,7.41)	0.001	0.022
DBP	4	26.8	0.251	1.00 (0.98,1.02)	0.945	0.982
BMI	6	57.3	0.039	1.01 (0.96,1.06)	0.614	0.538
BUN	4	56.8	0.074	1.06 (0.89,1.27)	0.525	0.795
Gender male	4	68.0	0.025	1.01 (0.58, 1.76)	0.984	0.800
ALB	3	93.7	0.000	0.89 (0.71,1.12)	0.329	0.943

SBP, systolic blood pressure; DBP, diastolic blood pressure; HbA1c, glycosylated haemoglobin; FBG, fasting blood glucose; BUN, blood urea nitrogen; eGFR, estimated glomerular filtration rate; UA, uric acid; Cr, creatinine; BMI, body mass index; ALB, albumin; DR, diabetic retinopathy; WHR, waist-to-hip ratio.

#### 3.2.1 SBP and hypertension

**Figure 2:**
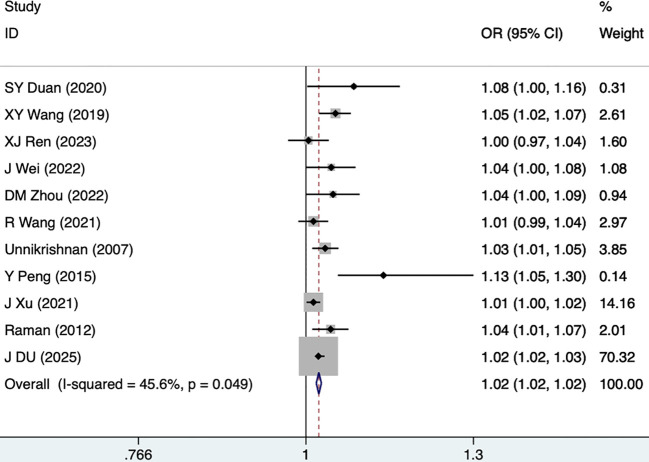
Forest plots for SBP.

**Figure 3:**
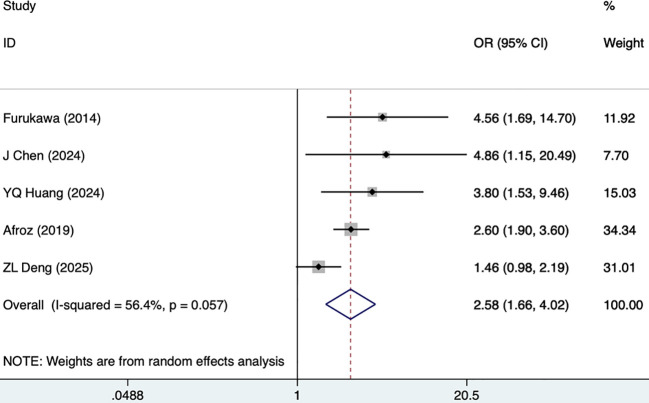
Forest plots for hypertension.

Eleven studies, including 5,631 participants, reported SBP as a risk factor for DKD. The fixed-effects model was utilized (I^2^ = 45.6%, P = 0.049). As shown in **Figure 2**, **Table 3**, SBP was demonstrated as a risk factor for DKD in T2DM (OR [95% CI]=1.02 [1.02-1.02], P<0.001). Hypertension was reported as a risk factor for DKD in five studies, including 3,319 participants, and a random-effects model was utilized (I^2^ = 56.4%, P = 0.057). As illustrated in **Figure 3**, **Table 3**, hypertension was demonstrated as a high-risk factor for DKD in T2DM (2.58 [1.66-4.02], P<0.001).

#### 3.2.2 FBG and HbA1c

**Figure 4:**
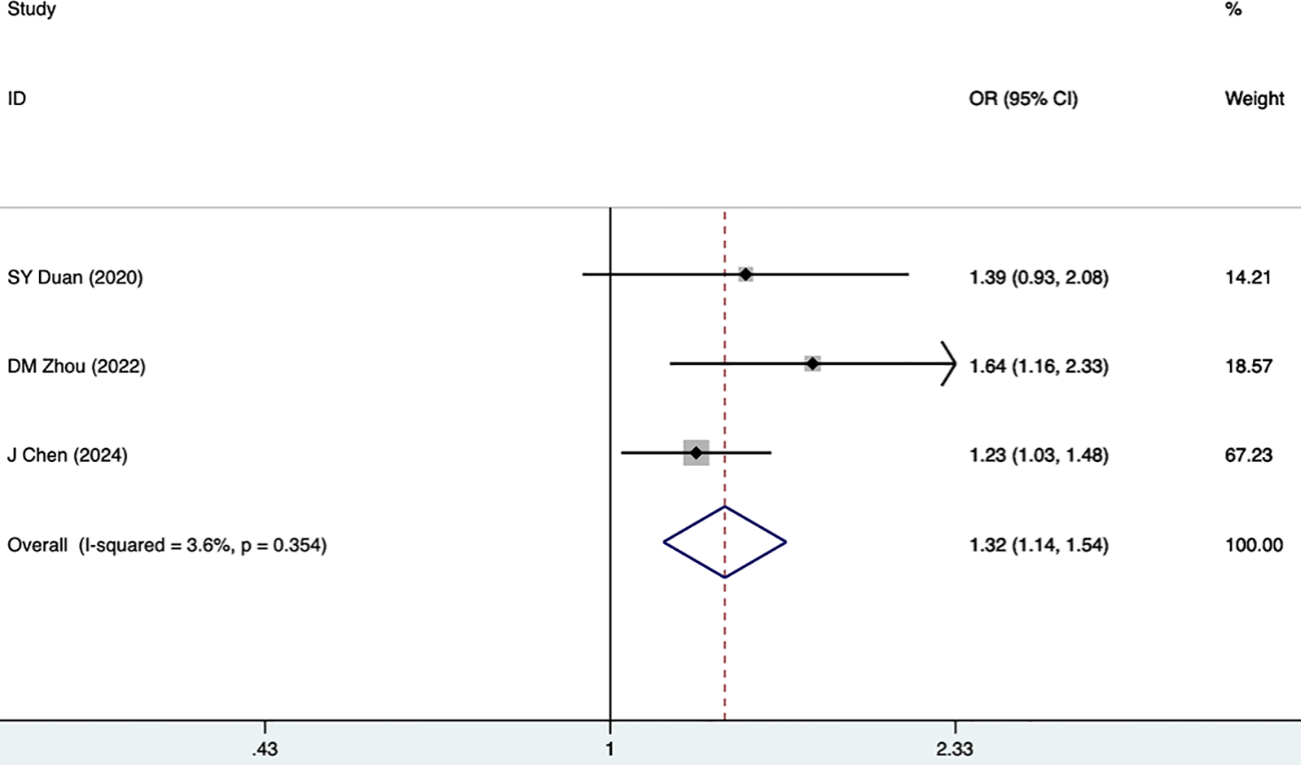
Forest plots for FBG.

**Figure 5:**
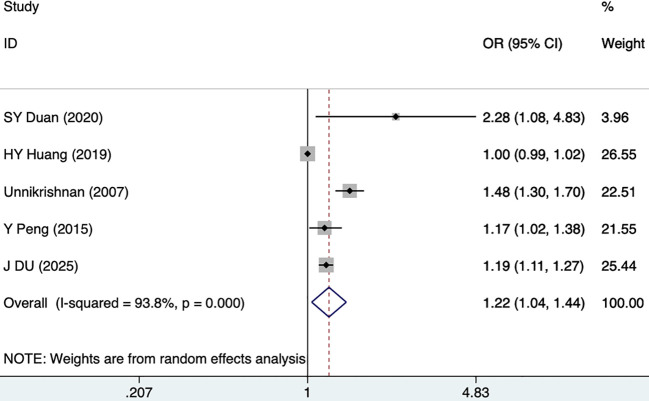
Forest plots for HbA1c.

Three studies, including 343 participants, mentioned the association between FBG and DKD. The fixed-effects model was utilized (I^2^ = 3.6%, P = 0.354). As shown in **Figure 4**, **Table 3**, FBG was a risk factor for DKD (1.32 [1.14-1.54], P<0.001). HbA1c was reported as a risk factor for DKD in five studies, including 4,188 participants. The random-effects model was utilized, given high heterogeneity (I^2^ = 93.8%, P = 0.000). As illustrated in **Figure 5**, **Table 3**, HbA1c was a risk factor for DKD (1.22 [1.04-1.44], P = 0.015).

#### 3.2.3 UA

**Figure 6:**
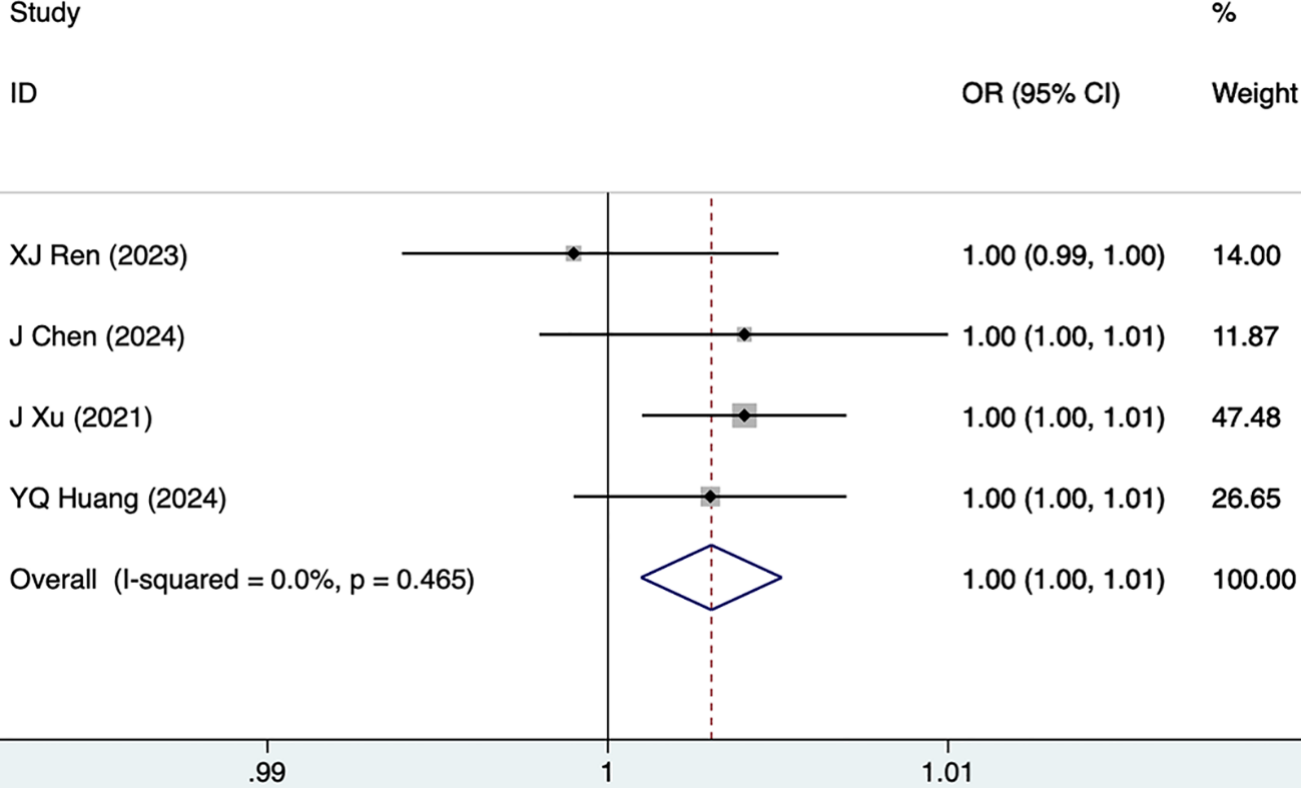
Forest plots for UA.

UA was reported as a risk factor for DKD in four studies, including 927 participants. The fixed-effects model was utilized (I^2^ = 0.0%, P = 0.465). As shown in **Figure 6**, **Table 3**, UA was a risk factor for DKD (1.00 [1.00-1.01], P = 0.004).

#### 3.2.4 Cr

**Figure 7:**
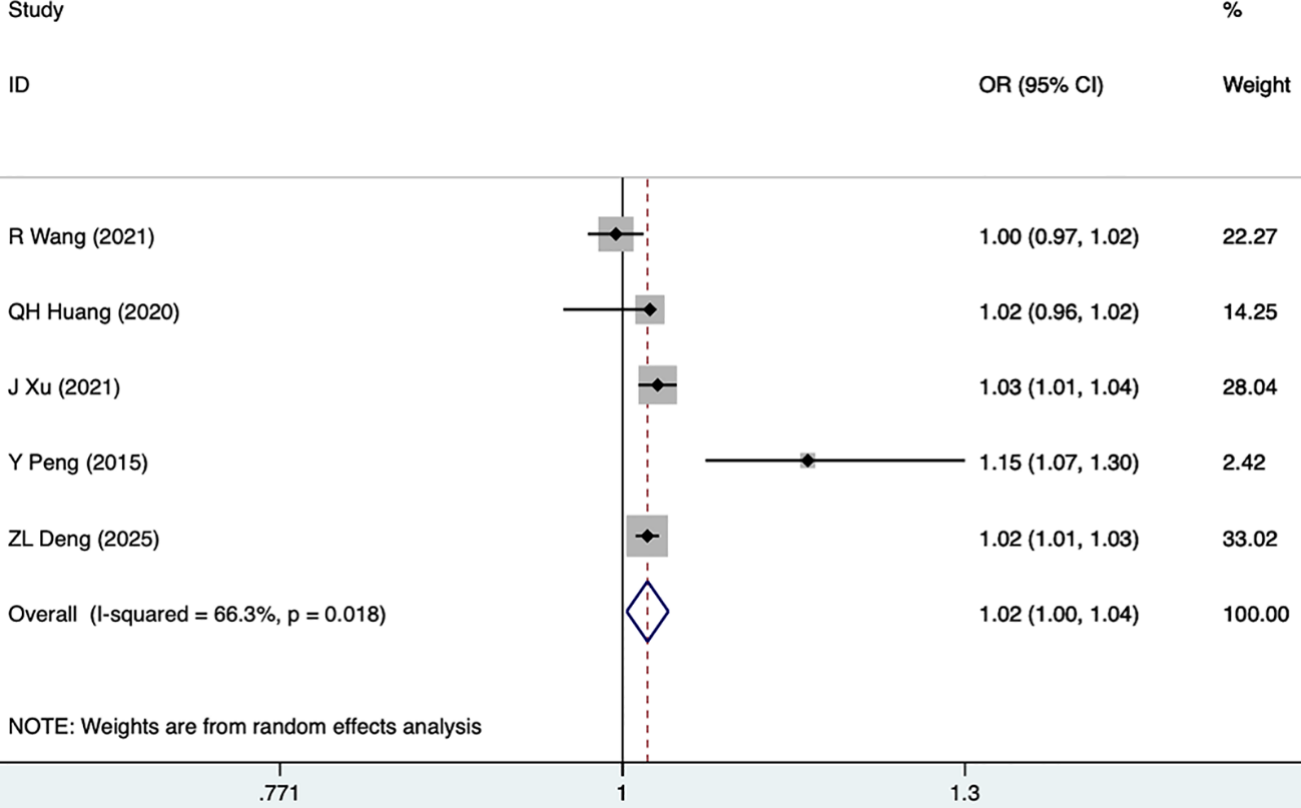
Forest plots for Cr.

Cr was reported as a risk factor for DKD in five studies, including 2,625 participants. The random-effects model was utilized (I^2^ = 66.3%, P = 0.018). As shown in **Figure 7**, **Table 3**, Cr was a risk factor for DKD (1.02 [1.00-1.04], P = 0.019).

#### 3.2.5 Age

**Figure 8:**
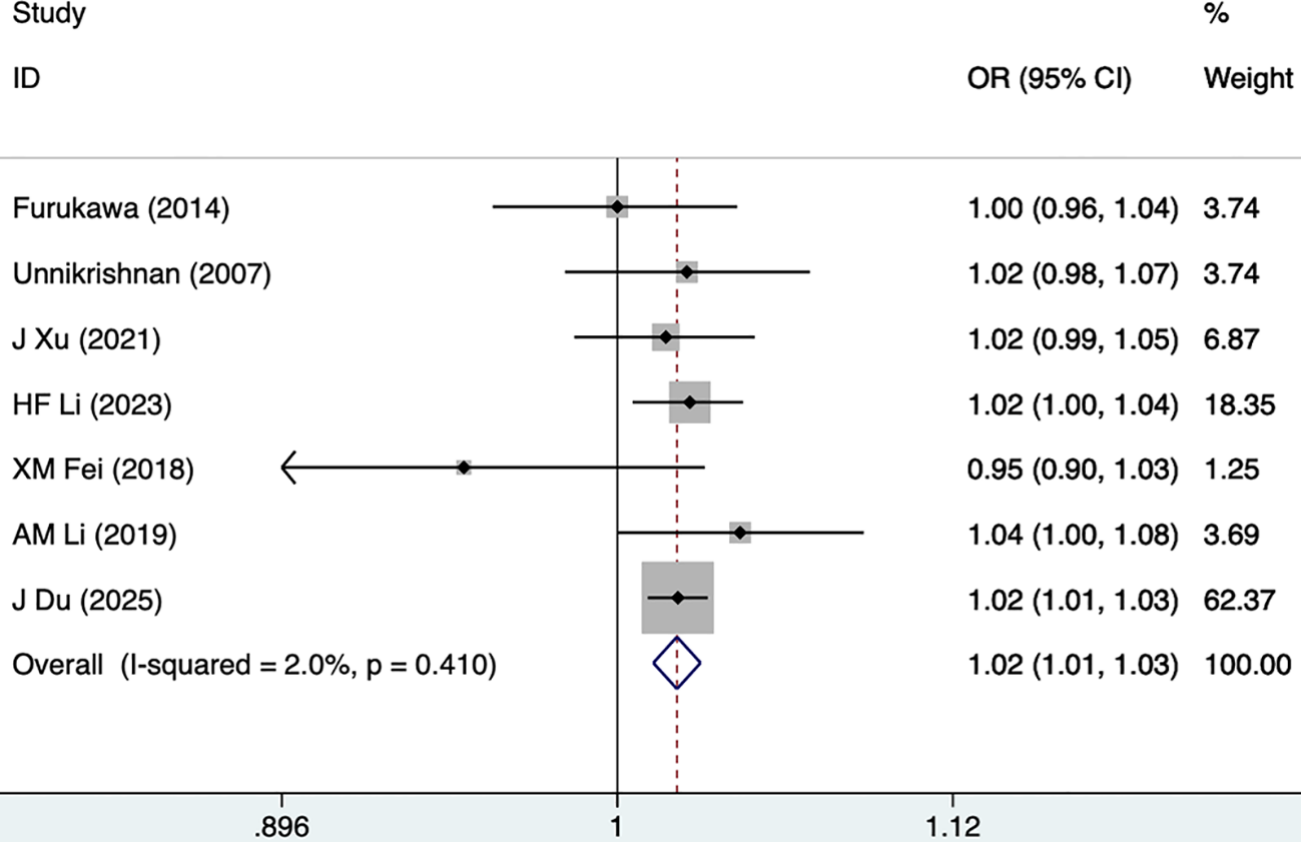
Forest plots for age.

Seven studies, including 5,623 participants, mentioned age as a risk factor for DKD, and the fixed-effects model was utilized (I^2^ = 2.0%, P = 0.410). As shown in **Figure 8**, **Table 3**, age was found as a risk factor for DKD (1.02 [1.01-1.03], P<0.001).

#### 3.2.6 Diabetes duration

**Figure 9:**
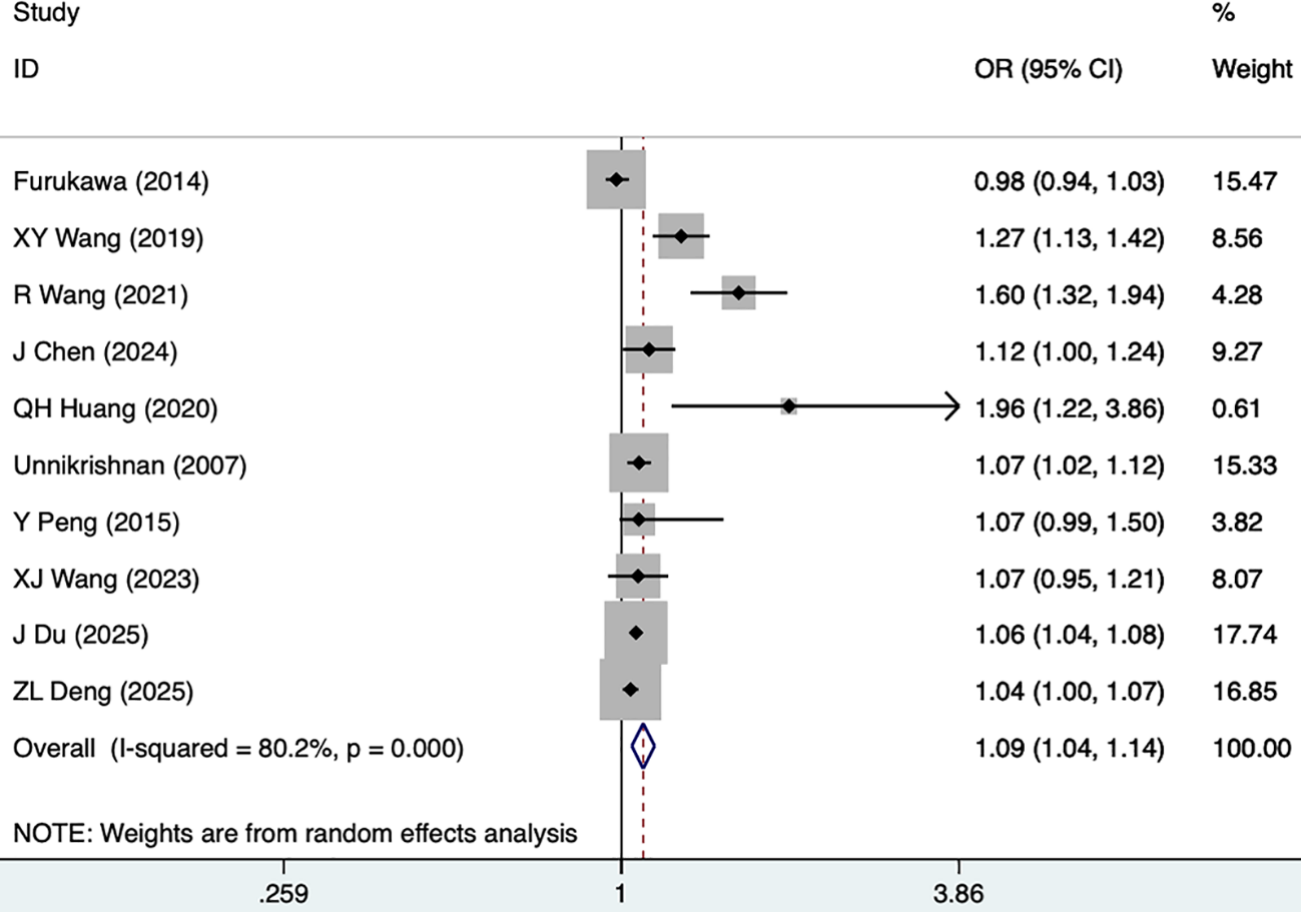
Forest plots for diabetes duration.

Diabetes duration was reported as a risk factor for DKD in ten studies, including 6,714 participants. The random-effects model was utilized, given high heterogeneity (I^2^ = 80.2%, P = 0.000). As illustrated in **Figure 9**, **Table 3**, diabetes duration was a risk factor for DKD (1.09 [1.04-1.14], P<0.001).

#### 3.2.7 DR

**Figure 10:**
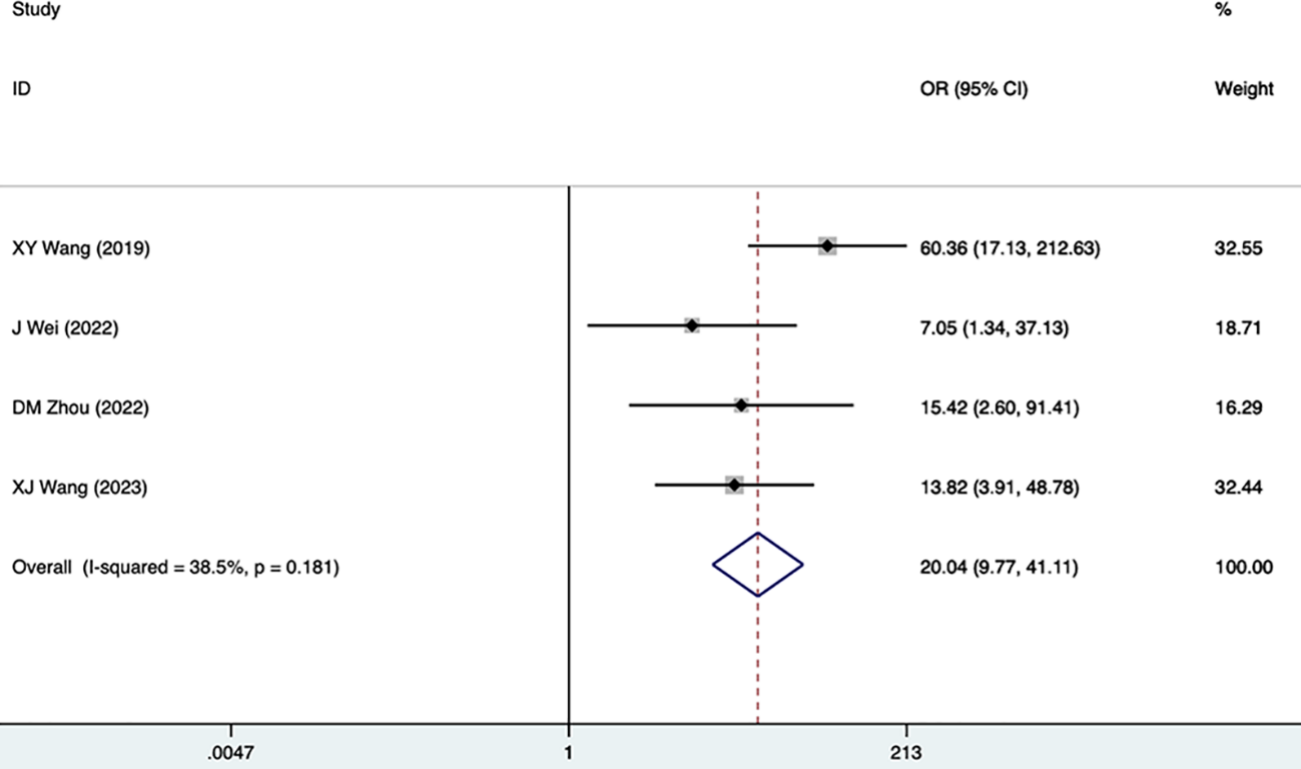
Forest plots for DR.

Four studies, including 513 participants, mentioned DR as a risk factor for DKD. The fixed-effects model was utilized (I^2^ = 38.5%, P = 0.181). As shown in **Figure 10**, **Table 3**, DR was strongly associated with DKD (20.04 [9.77,41.11], P<0.001).

#### 3.2.8 WHR

**Figure 11:**
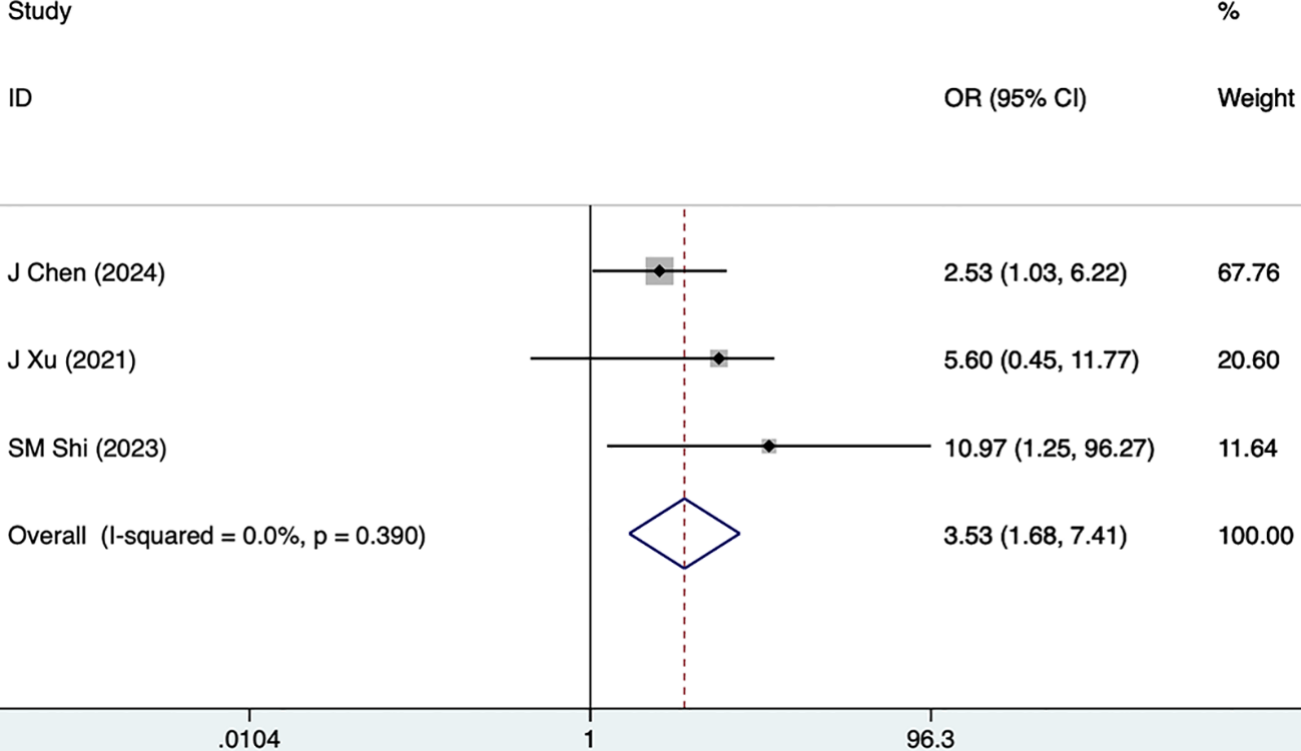
Forest plots for WHR.

WHR was reported as a risk factor for DKD in three studies, including 1685 participants, and the fixed-effects model was utilized (I^2^ = 0.0%, P = 0.390). As illustrated in **Figure 11**, **Table 3**, WHR was a risk factor for DKD (3.53 [1.68-7.41], P = 0.001).

#### 3.2.9 Others

Based on the available evidence from existing studies, this meta-analysis did not detect a statistically significant association of DBP (1.00 [0.98-1.02], P = 0.945), BMI (1.01 [0.96-1.06], P = 0.614), BUN (1.06 [0.89-1.27], P = 0.525), male gender (1.01 [0.58-1.76], P = 0.984) and ALB (0.89 [0.71-1.12], P = 0.329) with DKD (**Table 3**).

### 3.3 Sensitivity analysis

Because of significant heterogeneity, sensitivity analysis was conducted on diabetes duration (**Supplementary Figure 1A**), Cr (**Supplementary Figure 1B**), HbA1c (**Supplementary Figure 1C**), and hypertension (**Supplementary Figure 1D**). As demonstrated in **Supplementary Figures 1A, B**, the results for diabetes duration and Cr were stable. The study by HY Huang (25) may be the source of heterogeneity in HbA1c (**Supplementary Figure 1C**), and the study by ZL Deng (42) may be the source of heterogeneity in hypertension (**Supplementary Figure 1D**).

### 3.4 Subgroup analysis

#### 3.4.1 Clinical diagnosis subgroup

A total of 12 related factors, including SBP, age, diabetes duration, hypertension, UA, BMI, WHR, BUN, DBP, Cr, male gender and HbA1c for DKD were analyzed. Among them, 7 risk factors for DKD were identified. SBP was mentioned as a risk factor for DKD in six studies, including 3,341 participants, and the fixed-effects model was utilized (I^2^ = 46%, P = 0.099). SBP was found as a risk factor for DKD (1.02 [1.01-1.03], P<0.001) (**Supplementary Figure 2A**). Age was mentioned as a risk factor for DKD in six studies, including 3,832 participants, and the fixed-effects model was utilized (I^2^ = 18.2%, P = 0.296). Age was found as a risk factor for DKD (1.02 [1.01-1.03], P = 0.003) (**Supplementary Figure 2B**). Diabetes duration was reported as a risk factor for DKD in six studies, including 3504 participants. The random-effects model was utilized, given high heterogeneity (I^2^ = 85.1%, P = 0.000). Diabetes duration was found as a risk factor for DKD (1.14 [1.02-1.27], P = 0.016) (**Supplementary Figure 2C**). Hypertension was reported as a risk factor for DKD in four studies, including 2,270 participants, and the fixed-effects model was utilized (I^2^ = 0.0%, P = 0.583). Hypertension was found as a risk factor for DKD (2.87 [2.16-3.82], P<0.001) (**Supplementary Figure 2D**). BMI was mentioned as a risk factor for DKD in four studies, including 2218 participants, and the fixed-effects model was utilized (I^2^ = 42.2%, P = 0.158). BMI was found as a factor for DKD in T2DM (1.04 [1.00-1.08], P = 0.042) (**Supplementary Figure 2E**). Other risk factors, including UA and WHR, showed the same result as mentioned above (**Table 4**).

**Table 4:** Risk factors of clinical diagnostic group and pathological diagnostic group.

Risk factors	No. of study	Heterogeneity		OR (95%CI)	P	Egger
		I^2^ (%)	P			
Clinical diagnostic group						
SBP	6	46.0	0.099	1.02 (1.01, 1.03)	0.000	0.150
Age	6	18.2	0.296	1.02 (1.01,1.03)	0.003	0.234
Diabetes duration	6	85.1	0.000	1.14 (1.02,1.27)	0.016	0.103
Hypertension	4	0.0	0.583	2.87 (2.16,3.82)	0.000	0.008
UA	4	0.0	0.465	1.00 (1.00,1.01)	0.004	0.426
BMI	4	42.2	0.158	1.04 (1.00,1.08)	0.042	0.973
WHR	3	0.0	0.39	3.53 (1.68,7.41)	0.001	0.022
BUN	4	56.8	0.074	1.06 (0.89,1.27)	0.525	0.795
DBP	4	26.8	0.251	1.00 (0.98,1.02)	0.945	0.982
Cr	4	74.7	0.008	1.02 (0.99,1.05)	0.114	0.557
Gender male	4	68.0	0.025	1.01 (0.58, 1.76)	0.984	0.800
HbA1c	3	94.6	0.000	1.20 (0.93,1.54)	0.166	0.291
Pathological diagnostic group						
SBP	4	0.0	0.863	1.05 (1.03, 1.07)	0.000	0.442
DR	4	38.5	0.181	20.04 (9.77,41.11)	0.000	0.434

#### 3.4.2 Pathological diagnostic group

Two related factors, including SBP and DR, were analyzed, and both were risk factors for DKD. SBP was mentioned as a risk factor for DKD in four studies, including 481 participants and the fixed-effects model was utilized (I^2^ = 0.0%, P = 0.863) (**Supplementary Figure 2F**; **Table 4**). SBP was found as a risk factor for DKD (1.05 [1.03-1.07], P<0.001) in the pathological diagnosis group (**Supplementary Figure 2F**). DR was also found as a risk factor, with the same result as mentioned above (**Table 4**).

### 3.5 Publication bias

Egger’s test indicated that 8 risk factors had no publication bias (P > 0.05) (**Table 3**). Publication bias in HbA1c and WHR may be ascribed to the unclear risk of bias in the blinding of patients and personnel. The publication bias of articles incorporating various risk factors for DKD is presented in **Supplementary Figure 3**. Among these, the funnel plots for age and UA were symmetrical, indicating a minimal publication bias in these studies. However, the scatter points were concentrated in the lower part of the funnel plots, which may be due to small sample sizes. The funnel plots for the other risk factors were asymmetrical, suggesting a certain degree of publication bias in these studies.

## 4 Discussion

DKD has gradually replaced chronic glomerulonephritis in developing countries to become the primary cause of ESRD. The etiology of DKD is intricate and remains to be elucidated. It is necessary to understand modifiable risk factors that contribute to renal damage in diabetes patients in Asia. Although some studies have reported various risk factors for DKD, including HbA1c, fibrinogen, and cholesterol (44–46), this is the first meta-analysis to evaluate conventional risk factors for DKD in individuals with T2DM based on different diagnostic patterns in Asia. A total of 12,056 participants were enrolled, and 3,401 patients were diagnosed with DKD in this study. Totally, 15 related factors in 24 studies were included, and SBP, hypertension, WHR, FBG, HbA1c, UA, Cr, age and diabetes duration were risk factors of DKD in T2DM. DR was closely associated with DKD, and this association was also evident in subgroups defined by pathological diagnosis. SBP was a risk factor in both the clinical diagnosis group and the pathological diagnosis group. Our results are in accordance with the risk factors reported in the current ADA and KDIGO guidelines, including obesity, and hypertension (47). Compared with previous meta-analyses (14, 15, 48), we searched articles using different databases and performed subgroup analysis based on distinct diagnostic criteria for DKD to validate the accuracy of the findings. We focused primarily on traditional risk factors, which are more modifiable than genetic or environmental factors, thereby providing guidance for patients with diabetes. However, given the relatively small number of included studies and limited sample size, the incremental value of quantitative analysis may be limited, thus impacting the reliability of the conclusions.

Hypertension is a key factor in the progression of cardiovascular diseases in DKD patients. Although their causal relationship remains controversial, hypertension is regarded as a conventional risk factor for DKD (49–51). As early as 1997, an association was reported between hypertension and DKD in individuals with T2DM (52). Long-term hypertension can lead to glomerulosclerosis, renal tubulointerstitial fibrosis, and renal arteriosclerosis (53). Our analysis demonstrated a significant effect of hypertension on DKD, especially SBP, which was a risk factor in both the clinical diagnosis group and the pathological diagnosis group. This finding further confirms a significant association between SBP and DKD. T2DM is more common in middle-aged and older adults, whose hypertension mainly manifests as increased SBP. In an experimental model of Zucker rats with T2DM, obesity, and hypertension, SBP is tightly associated with glomerulosclerosis (51). Although lowering blood pressure is effective in hindering the progression of albuminuria in diabetes patients (54), tight control of blood pressure may induce hypotension and AKI (55) and increase the risk of all-cause mortality and cardiovascular mortality (56). Although the American Diabetes Association guidelines recommend a blood pressure target of <130/80 mmHg, and the Kidney Disease: Improving Global Outcomes guidelines suggest a more stringent systolic blood pressure target of <120 mmHg (47), developing individualized treatment plans is necessary.

WHR is a valuable indicator for assessing central obesity. While BMI is commonly employed to assess overweight or obesity, its accuracy is influenced by both muscle and bone mass. This study revealed that WHR was associated positively with DKD. However, this study did not detect a statistically significant association between BMI and DKD. A study has demonstrated that the AUC of model WHR for predicting the risk of DKD is larger than that of BMI in the Chinese individuals with T2DM (57). Therefore, we suggest that abdominal obesity is more likely to induce DKD by exacerbating insulin resistance, inflammation, endothelial dysfunction, fibrosis, arteriosclerosis, and thrombosis (58–61). Thus, maintaining a healthy weight and reducing fat accumulation, especially abdominal fat, is crucial for preventing DKD.

Hyperglycemia is a risk factor for DKD (62). HbA1c and FBG are frequently employed in clinical settings as primary indicators for controlling blood glucose in diabetes patients. Our analysis identified FBG and HbA1c as risk factors for DKD. However, due to the high heterogeneity in Hb1Ac, this conclusion should be interpreted with caution. Sensitivity analysis revealed that the heterogeneity of HbA1c may be attributable to the study by HY Huang (25). This may be related to the considerable differences in adjusted covariates between the study by HY Huang (25) and other studies. Numerous clinical studies have demonstrated that effective management of blood glucose levels significantly reduces the risk of microvascular complications among individuals with diabetes (63). Previous evidence has indicated that hyperglycemia can induce renal damage and ischemia through the activation of aldose reductase (64). Poor long-term control of blood glucose levels can lead to mesangial expansion, thickening of the glomerular basement membrane, and tubulointerstitial fibrosis, ultimately resulting in a decline in renal function (10, 65). Although the conclusion that HbA1c is a risk factor for DKD should be interpreted with caution, active control of blood glucose levels is crucial in the prevention of DKD.

In addition to HbA1c, diabetes duration and ALB also exhibited a significant heterogeneity (I^2^ >75%). Ten studies were included in the analysis of diabetes duration. The significant heterogeneity is likely multifactorial, arising from the mixed study types (retrospective or prospective study), disparate follow-up periods (1–4 years), and the varying reliability of patients’ recall of their disease onset. Three studies were included in the analysis of ALB. Heterogeneity is likely attributable to mixed study types (case-control or cohort study), divergent covariates, and distinct laboratory assays for ALB quantification. Thus, the results of outcomes with high heterogeneity should be interpreted with caution. More research is needed to validate these findings.

This analysis indicated that age and diabetes duration may be strong predictors of DKD. The risk of DKD exhibits an upward trend as individuals grow older, in accordance with previous results (50, 66). A retrospective chart review study demonstrates that for each one-unit elevation in age, the probability of developing DKD increases by 7.8% (67). This study further substantiated that a longer diabetes duration was associated with an increased prevalence of DKD. Al-Rubeaan et al. have revealed that diabetes duration, especially ≥15 years, is a crucial risk factor for DKD (68). Hence, screening for renal damage is essential in older patients with a longer duration of T2DM.

UA has been extensively described as a potential risk factor for hypertension, diabetes, stroke, and cardiovascular diseases (69–71). Our study also identified UA as a risk factor for DKD. UA possesses inflammatory properties and may contribute to endothelial dysfunction, thereby leading to DKD (72). UA may directly induce renal inflammation by depositing microcrystals in the lumen of the collecting duct, thereby contributing to intrarenal inflammation, interstitial fibrosis, albuminuria, and chronic kidney disease (73, 74). In a linear dose-response analysis, Ji et al. have discovered that the risk of DKD increases by 24% for each 1 mg/dl elevation in UA (75). However, it does not mean that a lower UA level reflects a lower risk of DKD. A prospective cohort study reports that in T2DM patients, both low and high serum UA levels are associated with the risk of the progression of albuminuria, regardless of potential confounding factors (76). Hence, maintaining UA within a specific concentration range helps prevent DKD in diabetic patients.

DR is another prevalent microvascular complication of diabetes. Its major pathological features are the proliferation of endothelial cells and the thickening of the basement membrane, leading to capillary blockage, visual impairment, and final blindness. The pooled OR for DR was unusually large, likely influenced by confounding factors (such as diabetes duration, glycemic exposure) and selection bias (biopsy-based cohorts). DR was more plausibly a marker of advanced microvascular disease rather than a causal risk factor. It can also demonstrate a strong association between the microvascular pathological changes in different target organ injuries caused by T2DM. It has been shown that deep learning algorithms based on retinal images may serve as an auxiliary tool for screening DKD in diabetes patients (77). Therefore, it is recommended that patients with DR undergo regular screenings for diabetic renal injury.

### 4.1 Strengths and limitations

Several strengths should be noted. This is the first meta-analysis of risk factors by classifying patients into different groups based on their diagnostic patterns. All included articles were of high quality. Detailed data on each risk factor were provided.

There are some limitations in this study. First, only English-language publications were included. Second, this meta-analysis focused on traditional risk factors for DKD, but did not explore gene-related risk factors, which are vital for understanding the mechanism of DKD. Third, the inclusion of both case-control and cohort studies may introduce heterogeneity and potential bias due to differences in study design. Thus, more comprehensive studies are needed to address these limitations, such as including both English-language and non-English publications, enrolling patients from diverse ethnicities and regions, and integrating traditional and gene-related risk factors. Such research will enhance the understanding of DKD in T2DM, providing more reliable evidence for clinical practice and global prevention of DKD.

## 5 Conclusions

This meta-analysis identifies several risk factors for DKD in T2DM. SBP, hypertension, HbA1c, WHR, FBG, UA, Cr, age and diabetes duration are found as risk factors for DKD in Asia. A close association is observed between DR and DKD, and this association remains consistent across subgroups based on pathological diagnosis. SBP is identified as a risk factor in both the clinical diagnosis and pathological diagnosis groups. These findings lay the groundwork for establishing prediction models for DKD. Further research on genetic risk factors for DKD is necessary.

## Data Availability

The original contributions presented in the study are included in the article/**Supplementary Material**. Further inquiries can be directed to the corresponding author.

## Supplementary material

Supplementary Figure 1 Sensitivity analysis for **(A)** diabetes duration; **(B)** Cr; **(C)** HbA1c; **(D)** hypertension.

Supplementary Figure 2 Subgroup analysis in Clinical diagnostic group of **(A)** SBP; **(B)** age; **(C)** diabetes duration; **(D)** hypertension; **(E)** BMI; **(F)** Subgroup analysis in Pathological diagnostic group of SBP.

Supplementary Figure 3 Funnel plots for **(A)** SBP, **(B)** hypertension, **(C)** FBG, **(D)** HbA1c, **(E)** UA, **(F)** Cr, **(G)** age, **(H)** diabetes duration, **(I)** DR, **(J)** WHR.

Supplementary Table 1 The quality of involved studies evaluated by NOS.
