# Supplementary material for: Risk factors for diabetic kidney disease in type 2 diabetes mellitus in Asia: a meta-analysis
Source: Front Endocrinol (Lausanne). 2026 Apr 14;17:1703533. doi: 10.3389/fendo.2026.1703533 (PMC13120950; doi:10.3389/fendo.2026.1703533)
Supplement: Supplementary Figure 1 — Sensitivity analysis for (A) diabetes duration; (B) Cr; (C) HbA1c; (D) hypertension. [file DataSheet1.docx]

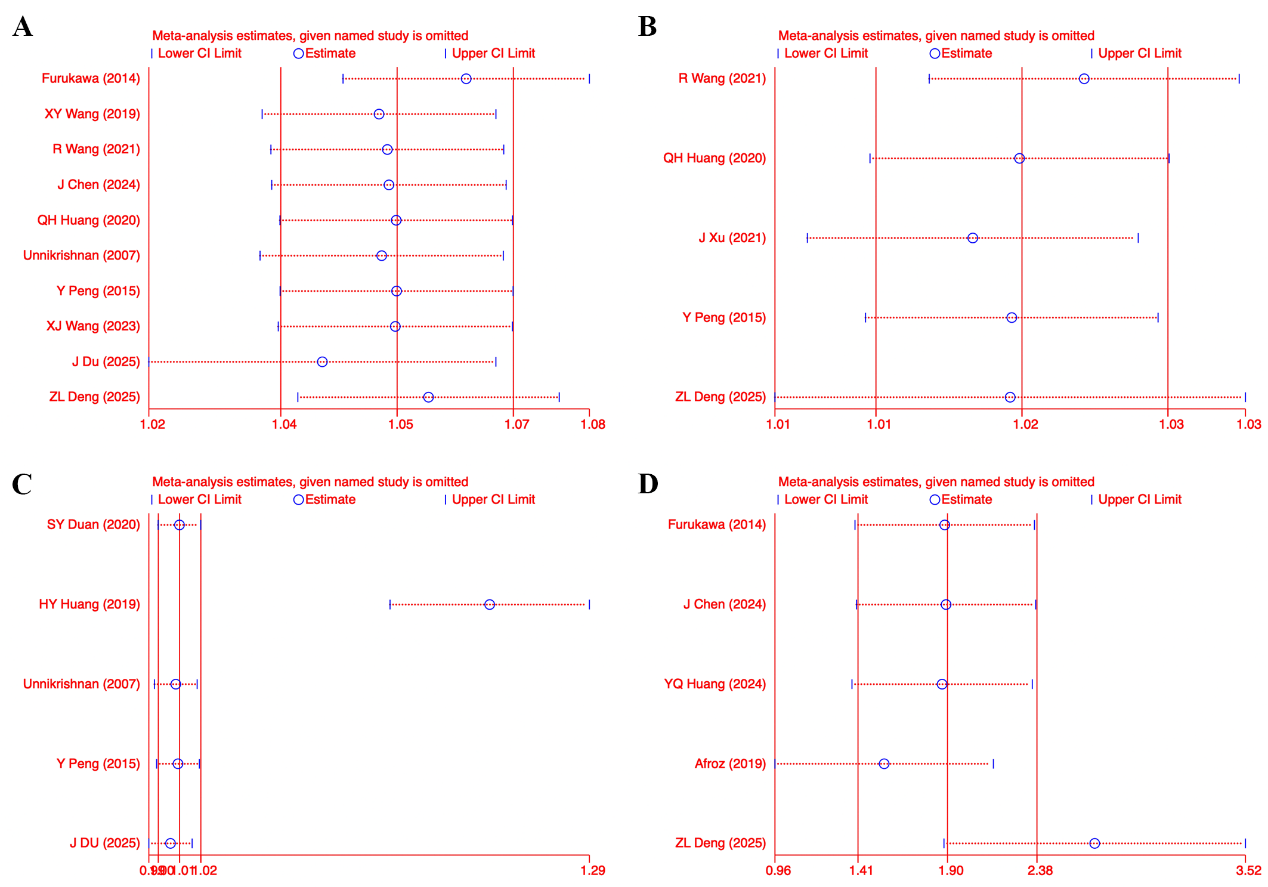


**Figure S1.** Sensitivity analysis for (A) diabetes duration; (B) Cr; (C) HbA1c; (D) hypertension


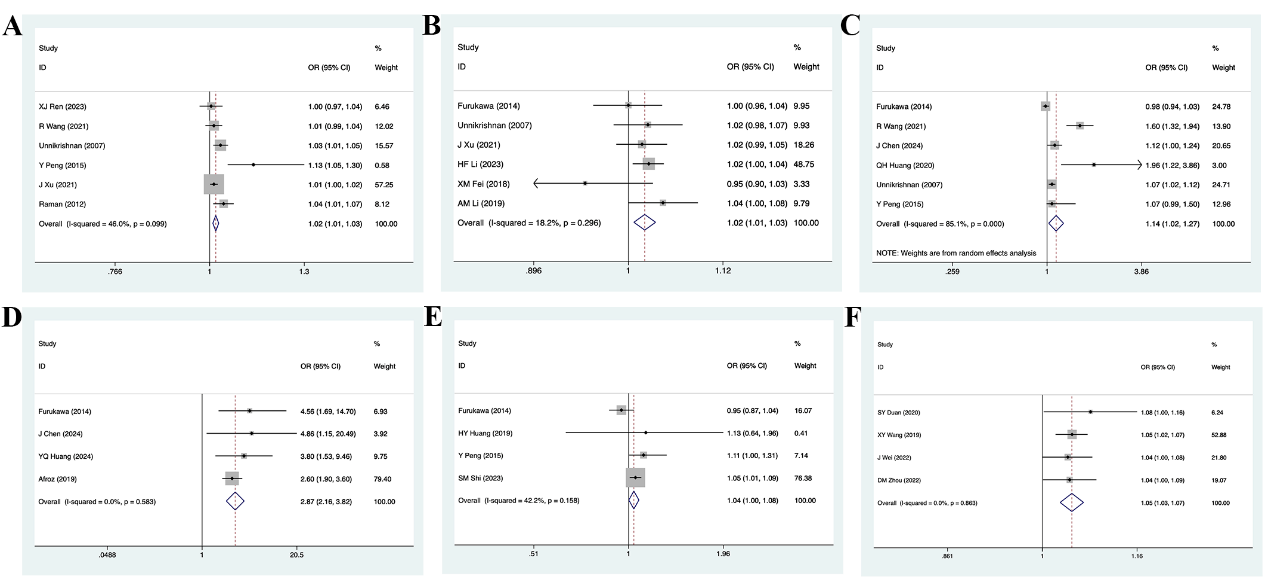


**Figure S2.** Subgroup analysis in Clinical diagnostic group of (A) SBP; (B) age; (C) diabetes duration; (D) hypertension; (E) BMI; (F) Subgroup analysis in Pathological diagnostic group of SBP


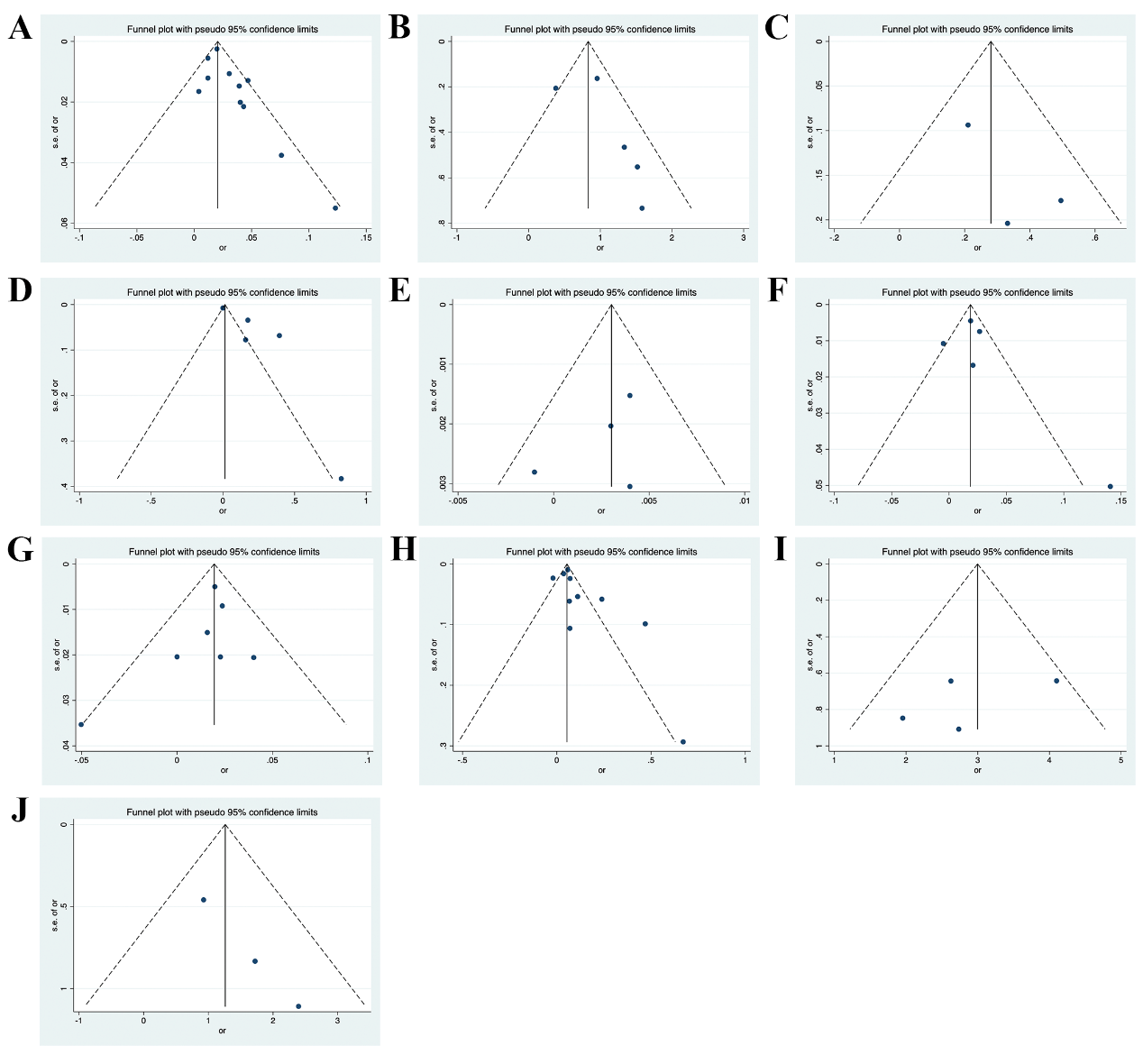


**Figure S3.** Funnel plots for (A) SBP, (B) hypertension, (C) FBG, (D) HbA1c, (E) UA, (F) Cr, (G) age, (H) diabetes duration, (I) DR, (J) WHR

**Table S1.** The quality of involved studies evaluated by NOS

| case control | | | | | | | | | |
| --- | --- | --- | --- | --- | --- | --- | --- | --- | --- |
| Study | Is the case definition adequate? | Representativeness of the cases | Determination of control group | Definition of Controls | Comparability of cases and controls based on the design or analysis | Ascertainment of exposure | Same method of ascertainment for cases and controls | Non response | Total scores |
| Furukawa  2014 | * | * | * | * | ** | * | * | * | 9 |
| T Li 2023 | * | * | * | * | ** | * | * | * | 9 |
| XJ Ren 2023 | * | * | * | * | * | * | * | * | 8 |
| YQ Huang 2023 | * | * | * | * | * | * | * | * | 8 |
| R Wang 2021 | * | * | * | * | * | * | * | * | 8 |
| QH Huang 2020 | * | * | * | * | * | * | * | * | 8 |
| J Du 2025 | * | * | * | * | ** | * | * | * | 9 |

| cohort study | | | | | | | | | |
| --- | --- | --- | --- | --- | --- | --- | --- | --- | --- |
| Study | Representativeness of the exposed group | Selection of non-exposed groups | Determination of exposure factors | Identification of outcome indicators not yet to be observed at study entry | Comparability of exposed and unexposed groups considered in design and statistical analysis | design and statistical analysis | Adequacy of the study's evaluation of the outcome | Adequacy of follow-up in exposed and unexposed groups | Total scores |
| SY Duan 2020 | * | * | * | ** | * | * | * | * | 9 |
| HY Huang 2019 | * | * | * | ** | * | * | * | * | 9 |
| SM Shi  2023 | * | * | * | * | * | * | * | * | 8 |
| XY Wang  2019 | * | * | * | ** | * | * | * | * | 9 |
| J Wei  2022 | * | * | * | ** | * | * | * | * | 9 |
| DM Zhou  2022 | * | * | * | * | * | * | * | * | 8 |
| J Chen  2024 | * | * | * | ** | * | * | * | * | 9 |
| Afroz  2019 | * | * | * | ** | * | * | * | * | 9 |
| Raman  2012 | * | * | * | ** | * | * | * | * | 9 |
| Unnikrishnan  2007 | * | * | * | * | * | * | * | * | 8 |
| Y Peng 2015 | * | * | * | * | * | * | * | * | 8 |
| J Xu 2021 | * | * | * | * | * | * | * | * | 8 |
| XJ Wang 2023 | * | * | * | ** | * | * | * | * | 9 |
| HF Li 2023 | * | * | * | * | * | * | * | * | 8 |
| XM  Fei 2018 | * | * | * | ** | * | * | * | * | 9 |
| AM  Li 2019 | * | * | * | ** | * | * | * | * | 9 |
| ZL Deng 2025 | * | * | * | ** | * | * | * | * | 9 |
